# Physiologically-based modelling in mice suggests an aggravated loss of clearance capacity after toxic liver damage

**DOI:** 10.1038/s41598-017-04574-z

**Published:** 2017-07-24

**Authors:** Arne Schenk, Ahmed Ghallab, Ute Hofmann, Reham Hassan, Michael Schwarz, Andreas Schuppert, Lars Ole Schwen, Albert Braeuning, Donato Teutonico, Jan G. Hengstler, Lars Kuepfer

**Affiliations:** 1 Joint Research Center for Computational Biomedicine, RWTH Aachen University, Aachen, Germany; 2 Leibniz Research Centre for Working Environment and Human Factors at the Technical University Dortmund, Dortmund, Germany; 3 Department of Forensic Medicine and Toxicology, Faculty of Veterinary Medicine, South Valley University, Qena, Egypt; 4 Dr. Margarete Fischer-Bosch Institute of Clinical Pharmacology and University of Tuebingen, Stuttgart, Germany; 5 German Federal Institute for Risk Assessment, Dept. Food Safety, Berlin, Germany; 6 Systems Pharmacology, Bayer AG, Leverkusen, Germany; 7 Fraunhofer MEVIS, Bremen, Germany; 8 Clinical PK and Pharmacometrics, Institut de Recherches Internationales Servier, Suresnes, France

## Abstract

Diseases and toxins may lead to death of active liver tissue, resulting in a loss of total clearance capacity at the whole-body level. However, it remains difficult to study, whether the loss of metabolizing tissue is sufficient to explain loss of metabolic capacity of the liver or whether the surviving tissue undergoes an adaptive response to compensate the loss. To understand the cellular impact of toxic liver damage in an *in vivo* situation, we here used physiologically-based pharmacokinetic modelling to investigate pharmacokinetics of a specifically designed drug cocktail at three different sampling sites of the body in healthy mice and mice treated with carbon tetrachloride (CCl_4_). Liver zonation was explicitly quantified in the models through immunostaining of cytochrome P450s enzymes. Comparative analyses between the simulated decrease in clearance capacity and the experimentally measured loss in tissue volume indicated that CCl_4_-induced impairment of metabolic functions goes beyond the mere loss of metabolically active tissue. The here established integrative modelling strategy hence provides mechanistic insights into functional consequences of toxic liver damage in an *in vivo* situation, which would not have been accessible by conventional methods.

## Introduction

The liver is the organ with the highest detoxification capacity in mammals. Hepatocytes, the parenchymal cells of the liver, modify the structure of xenobiotics to improve excretion through urine or feces. Usually, this is achieved by oxidation or hydrolysis in phase I of xenobiotic metabolism, followed by glucuronidation, sulfation, acetylation or glutathione conjugation in phase II. The liver is organized in lobes, which consist of similarly built functional units, the lobules. Lobules receive blood from branches of the hepatic artery and the portal vein. Subsequently, the blood flows along sinusoids into central veins and leaves the liver through the hepatic vein. Hepatocytes are aligned along sinusoids, microvessels of the liver, from which they are separated by fenestrated endothelial cells and the space of Disse. Hepatocytes in the periportal zone (upstream, at the side of the portal vein and the hepatic artery) have a different enzymatic setup than hepatocytes in the pericentral zone (downstream, at the side of the central vein)^1^. This compartmentalization of parenchymal cells along the sinusoid, usually referred to as zonation, has a significant functional impact on hepatic metabolic capacity, including drug activation and detoxification. In drug metabolism, cytochrome P450 enzymes are mainly located in the pericentral area of the liver lobule, while glutathione peroxidase shows in turn a stronger expression in the periportal zone^2^. Diseases and toxins which specifically affect particular zones in the liver lobule may have a different functional impact on hepatic clearance capacity as toxins which unspecifically affect overall hepatic protein concentration^2^. For example, it has been reported that ferrous sulfate and phosphorus specifically induce periportal damage^3^, while carbon tetrachloride (CCl_4_) causes pericentral damage^4^. The impact of different toxins on the zonated hepatic glucose or nitrogen metabolism has been investigated before^5^. Despite the significant involvement of the zonated cytochrome P450 enzymes and phase II enzymes in drug metabolism^2^, it is still unknown, how and to which extent destruction of specific lobular zones influences the pharmacokinetics (PK) of xenobiotics.

**Figure 1:**
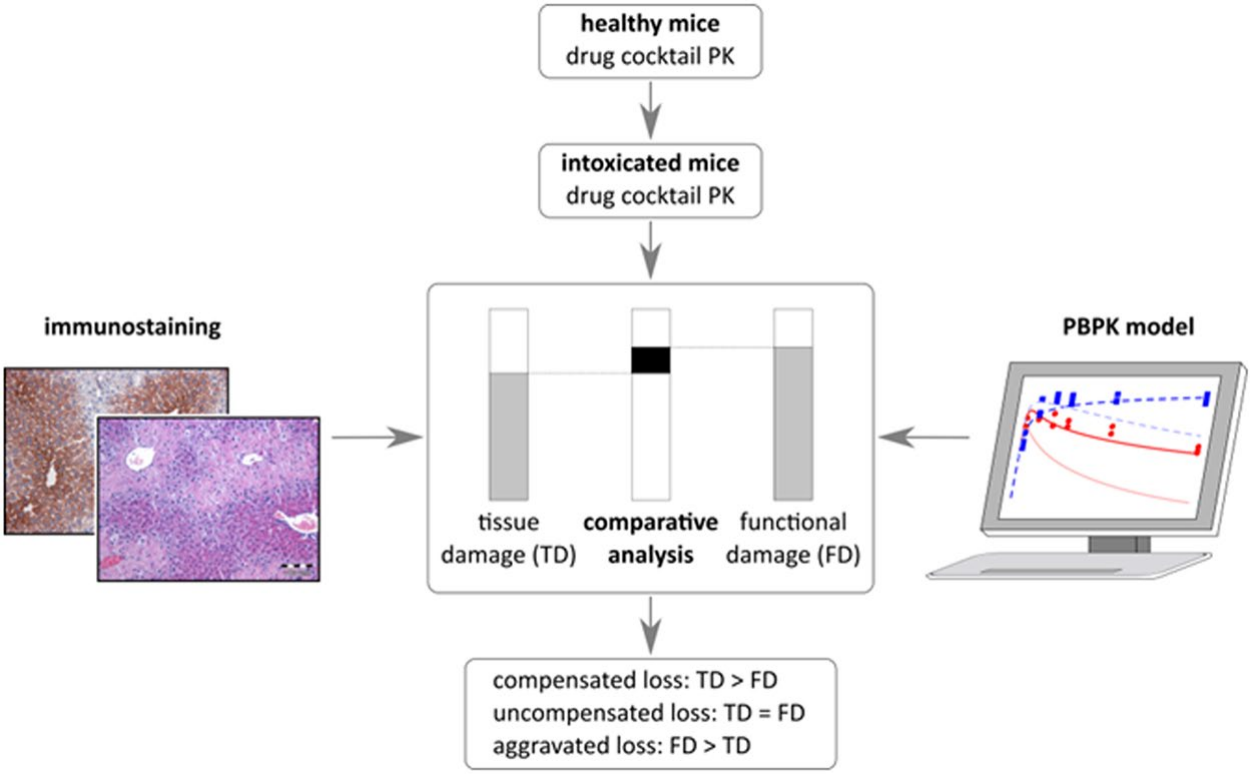
Overall workflow. In a first step, models for healthy mice were established on the basis of literature knowledge and own experimental data including PK measurements and quantification of enzyme expressing area of the liver lobule. The models were further adjusted to the plasma concentration profiles of intoxicated mice by reducing the clearance capacity of the liver (functional damage, FD). In a complementary approach, the dead cell area of the liver lobule was measured to quantify the tissue damage (TD). The functional damage was then compared to the tissue damage to differentiate between compensated, uncompensated and aggravated loss, respectively, of metabolically active tissue.

In this study, CCl_4_ intoxication was used as a model to investigate how toxin-induced liver damage of the pericentral zone affects drug PK in mice and in particular hepatic clearance capacity. To this end PK of the six parent drugs (caffeine, midazolam, torsemide, codeine, talinolol, pravastatin) and some of the corresponding metabolites were first measured and simulated at three sampling sites (right heart chamber, portal vein and hepatic vein) in healthy mice (Fig. 1). The same plasma concentration profiles were then determined for intoxicated mice 24 hours after CCl_4_ administration, the time point when destruction of liver tissue reaches a maximum. Physiologically-based pharmacokinetic (PBPK) modelling was used in order to quantify the functional damage following the loss of the pericentral compartment of the liver lobule by CCl_4_ intoxication.

In brief, PBPK modelling aims for a detailed mechanistic representation of physiological processes governing drug PK at the whole-body level^6^. In particular, the various compartments in PBPK models correspond explicitly to organs and tissues of the body. On the one hand, PBPK models are based on large-scale collections of physiological parameters such as organ volumes or surface areas, which are provided to the user by the modelling software itself. On the other hand, physicochemical properties of a compound such as lipophilicity or molecular weight are used to parametrize the distribution model describing the underlying mass balance in PBPK models. To specifically account for liver zonation, we also quantified the area of expression of four cytochrome P450 enzymes involved in the metabolism of the drug cocktail in mice, namely CYP1A, CYP2C, CYP3A and CYP2D.

In a complementary approach, the extent of CCl_4_-induced liver damage at tissue level was analyzed by hematoxylin and eosin staining (H&E-staining). A comparison between the functional damage and the tissue damage allowed us to draw further conclusions about the extent of CCl_4_-induced intoxication within the surviving liver tissue.

## Results

We started our analyses by describing the PK of the six drugs simultaneously administered as a bolus injection of the cocktail^7, 8^ with PBPK models. The composition of the administered drug cocktail (caffeine, midazolam, torsemide, codeine, talinolol, pravastatin) has been designed to selectively quantify the capacity of active clearance processes in humans^7^. In mice, the main metabolizing enzymes for caffeine, midazolam, torsemide, i.e., CYP1A, CYP3A, CYP2C, respectively, have been identified previously. However, the remaining orthologous enzymes or active transporters are unknown. Metabolism of caffeine, midazolam and torsemide results in the formation of three main metabolites paraxanthine, 1′-hydroxymidazolam and hydroxytorsemide, respectively. All three metabolites were determined experimentally in plasma samples of mice and were also considered in the PBPK simulations. In addition to plasma concentration profiles of the different compounds, enzyme availability was measured by immunostaining to locate the position of four metabolizing enzymes within the liver lobule.

**Figure 2:**
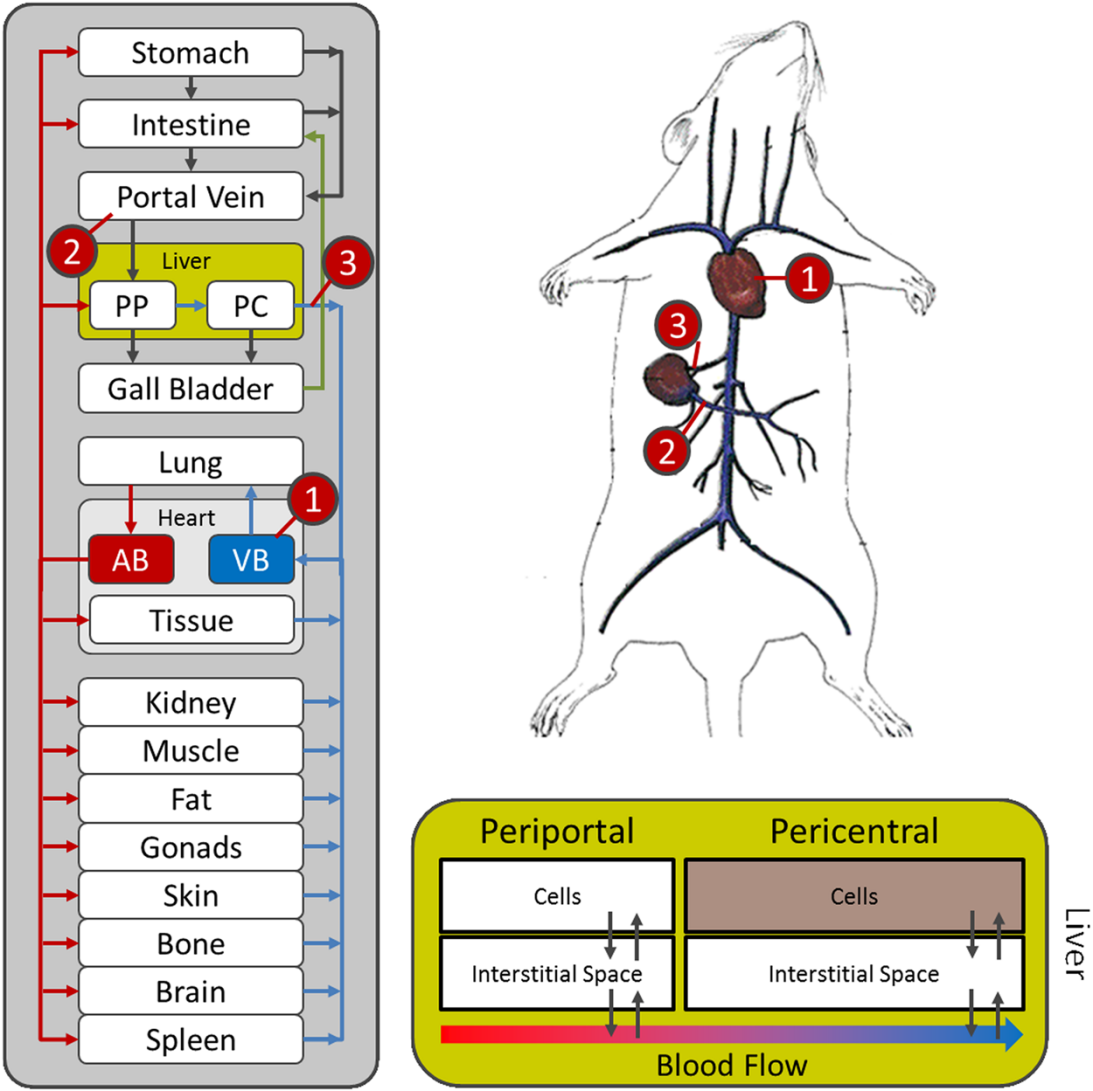
PBPK modelling in mice. Plasma concentration profiles from three different sampling sites where used in this study: the right heart chamber (1) and the portal vein (2) (both contributing to the liver inflow) and the hepatic vein (3) (representing the liver outflow). Due to the detailed structure of PBPK-models (left), it was possible to include all three sampling sites into the model at the same time (upper right). The volume fractions of the periportal and the pericentral zone of the liver lobule (lower right) were set according to the previously determined fractions of expression (Table 1).

The drug plasma concentration profiles were gathered by administering the drug cocktail to healthy mice. The overall dosage was sufficiently low to exclude the possibility of drug-drug interactions^7, 8^. Usually, drug plasma concentration profiles are only determined from samples in the venous blood as such allowing the quantification of total drug clearance at the whole-body level. However, an explicit quantification of liver clearance is difficult if only a single plasma compartment is considered. To allow an accurate quantification of hepatic clearance capacity and to pinpoint the overall mass balance at organism scale, we hence measured plasma concentration profiles from three different sampling sites within the body: (i) the right heart chamber, (ii) the portal vein, and (iii) the hepatic vein (*see* Suppl. Data). Since PBPK models describe the physiology of an organism at a large level of detail, it was possible to specifically map the experimental sampling sites to the model and to simultaneously consider all three concentration profiles (Fig. 2). Notably, the three sampling sites allow quantifying the hepatic as well as the extrahepatic contribution to drug metabolism. Moreover, the overall mass balance of the model is further confined by the consideration of additional data points from different sites of the body. From a general perspective, the liver inflow can be described as a mixture of the blood from the portal vein (80%) and the hepatic artery (20%), which is again supplied from the overall arterial blood pool^9^. Measured concentrations in the portal vein and the hepatic vein correspond directly to liver in- and outflow, respectively. However, the concentration in the right heart chamber is only an approximation for the inflowing hepatic artery, since lung passage downstream of the heart and distribution in the surrounding tissue might influence the actual drug profile in the liver. Here, the PBPK models provide a possibility to explicitly close the hepatic mass balance by simulating the concentration profile in the hepatic artery based upon measurements in the right heart chamber.

### Extension of PBPK models by including liver zonation

**Figure 3:**
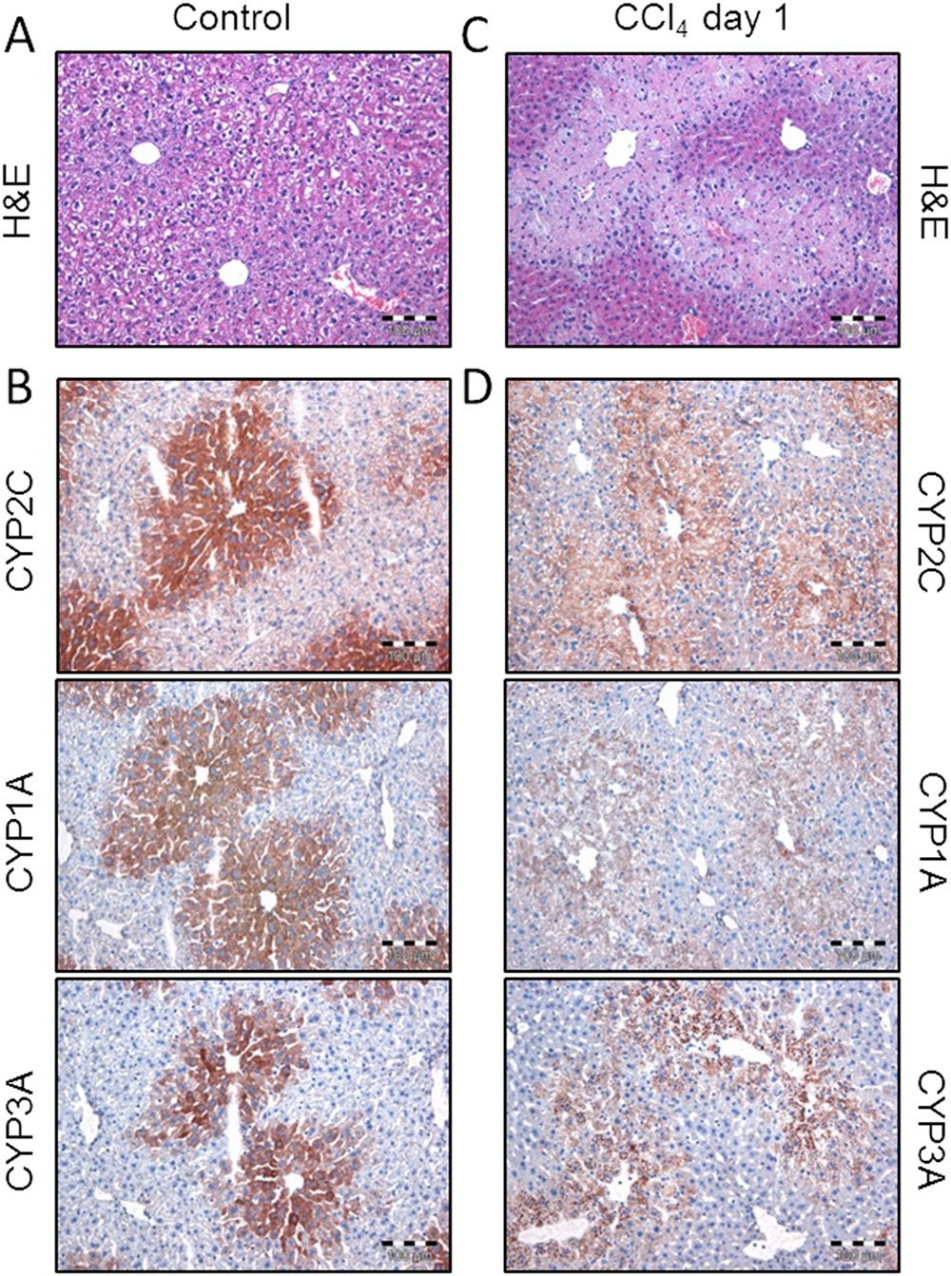
Staining of the liver lobule before and after CCl_4_ administration. (**A**) H&E staining in healthy mice. (**B**) Immunostaining of three cytochrome P450 enzymes (CYP1A, CYP3A and CYP2C) in healthy mice. (**C**) H&E staining in intoxicated mice. (**D**) Immunostaining of three cytochrome P450 enzymes (CYP1A, CYP3A and CYP2C) in intoxicated mice.

To enable a mechanistic representation of liver zonation in the models, we quantified the area of expression for CYP1A, CYP3A, CYP2C and CYP2D, the four cytochrome P450 enzymes involved in metabolism of caffeine, midazolam, torsemide and codeine, respectively (Fig. 3). For this purpose mouse liver slices were immunostained by antibodies directed against these enzymes and the positively stained area was quantified. All three enzymes showed a restricted pericentral expression pattern in the liver lobule. Image analysis revealed that CYP1A, CYP3A, CYP2C, CYP2D were expressed in 56%, 47%, 48% and 10% of the liver lobule, respectively (Table 1). To allow consideration of liver zonation we used a refined PBPK model where the liver lobule was divided into two parts, as such representing the periportal and pericentral zone (Fig. 2). The volumes of these zones were set relative to the values quantified by the image analyses described above. Generally, it may be possible that the expression of cytochrome P450 is heterogeneously distributed within the pericentral region. The bipartite differentiation between a periportal and pericentral zone hence leads to a certain degree of averaging. However, since the relative expression of ADME-associated enzymes and transporters (ADME: absorption, distribution, metabolization and excretion) is afterwards multiplied by the catalytic rate constant this simplification will finally have no substantial effect in the end.

**Table 1:** Relative pericentral expression (mean ± SD, N=3), functional damage and tissue damage for CYP1A, CYP3A, CYP2C and CYP2D, metabolizing caffeine, midazolam, torsemide and codeine, respectively.

Enzyme	Caffeine	Midazolam	Torsemide	Codeine
	CYsP1A	CYP3A	CYP2C	CYP2D
Pericentral expression	56 ± 4%	47 ± 8%	48 ± 1%	10 ± 1%*
**Functional damage**	98.3%	84%	92%	—
**Tissue damage**	65%	77%	75%	100%

^*^The staining data for CYP2D was not used in the analysis since the fraction of codeine which is metabolized through this enzyme is™ unknown.

Analogously to all other organs in the PBPK model, both the periportal and the pericentral zones are composed of the cellular space, the interstitium, the plasma and the red blood cells, respectively. The zones are linked by blood flow, as such accounting for mass transport in plasma and through red blood cells. Passive and active mass transfer only occurs between the different subcompartments of each zone. Notably, the zonated liver was only applied for those drugs for which the main metabolizing cytochrome P450 enzymes were known and for which PK measurements of a metabolite was available, i.e. caffeine, midazolame and torsemide. For codeine, talinolol and pravatstatin, a standard non-zonated liver model was used. An ubiquitous availability of hepatic transporter expression was also assumed for pravastatin and talinol, since the expression of organic anion transporting polypeptide 1 (OATP1) was found to be homogenously distributed between the periportal and the pericentral zone in humans^10^.

### PBPK modelling of the drug cocktail

For PBPK model development the available physicochemical information was first gathered from literature (Table 2). These include lipophilicity, molecular weight and fraction unbound for each of the six parent compounds. Note that this information is sufficient to parametrize the basic structure of the underlying PBPK distribution model, since the physiology of the organism, for example the organ volumes or blood flow rates between different organs are available in the PBPK modelling software itself^6^. Furthermore, compound-specific parameters in the model such as tissue permeabilities or organ-plasma partition coefficients are calculated based upon physicochemical information of the drug considered^6, 11^. In addition to the physicochemical properties, the active enzyme- and transporter-mediated clearance processes were considered (Table 3). The level of detail of such knowledge differs between the compounds. Likewise, while relative expression of ADME-associated enzymes and transporters can generally be quantified at the whole-body level^12^ only Cyp3a is available extrahepatically. To ensure a comparable complexity between the PBPK models of the different compounds, only hepatic expression of Cyp3a was considered in the PBPK model for midazolam as well. The following sections describe in detail how each model was created (Figures S1–S6). Notably, the parameters provided in Tables 2 and 3 are sufficient to specifically parametrize each of the PBPK models discussed in the following.

**Table 2:** Physicochemical parameters used in the PBPK models.

Substance	Dose [mg/kg]	Fu	Lipophilicity	Molecular weight [g/mol]
Caffeine	5	0.85^15^^,^***	−0.07	194.19
Paraxanthine	—	0.85	−0.63*	180.16
Midazolam	2	0.046^30^	3.33*	325.77
1′-Hydroxymidazolam	—	0.0125**	3.09*	341.77
Torsemide	2	0.01^31^^,^***	2.3	348.42
Hydroxytorsemide	—	0.01	0.75*	364.41
Codeine	2	0.7^31^^,^***	1.54**	299.36
Talinolol	1	0.39^31^^,^***	2.3*	363.49
Pravastatin	20	0.5^31^^,^***	1.65*	424.53

*Literature **optimized ***human value.

**Table 3:** Parameters of the active clearance processes.

Substance	Parameters
Caffeine	CL_Liver_ = 0.59 1/min; CL_renal_ = 0.0324 1/min
Paraxanthine	CL_Liver_ = 3.5E-7 1/min; CL_renal_ = 0.00926 1/min
Midazolam	1′-HM: k_cat_ = 80.82 1/min; K_m_ = 0.95 µmol/l; 4′-HM: k_cat_ = 28.73 1/min; K_m_ = 8.43 µmol/l
1′-Hydroxymidazolam	CL_Liver_ = 15.6 1/min
Torsemide	CL_Liver_ = 1.93 1/min; CL_renal_ = 0.64 1/min
Hydroxytorsemide	CL_Liver_ = 15.3 1/min; CL_renal_ = 79.6 1/min
Codeine	CL_Liver_ = 1.60 1/min; CL_renal_ = 0.21 1/min
Talinolol	CL_Liver_ = 2.95 1/min; CL_renal_ = 39.76 1/min
Pravastatin	CL_Liver_ = 2.52 1/min; CL_bilary_ = 0.00000457 1/min; CL_renal_ = 500 1/min

### Caffeine

Concentration profiles for both the parent drug caffeine and the metabolite paraxanthine were measured and considered for the establishment of the PBPK model for caffeine. 87% of the metabolism of caffeine is due to CYP1A2 at physiological concentrations^13^. First order clearance by CYP1A2 was introduced both for caffeine and for paraxanthine^14^. By immunostaining we found that CYP1A is pericentrally expressed in 56% of the liver lobule (Table 1). The relative formation rate of paraxanthine relative to the three other main metabolites theophylline, theobromine and trimethylurate is 27%^14^. 5.1% of the administered caffeine are excreted unchanged in the urine and 10.7% are secreted through paraxanthine^15^. This physiological information was additionally included for the development of the PBPK for caffeine and paraxanthine (Figure S1).

### Midazolam

Concentration profiles for both the parent drug midazolam and the major metabolite of midazolam, 1′-hydroxymidazolam, were measured and considered for the establishment of the PBPK model for midazolam. By immunostaining it was found that CYP3A is pericentrally expressed in 47% of the liver lobule (Table 1). While only concentration profiles of the major metabolite 1′-hydroxymidazolam was measured experimentally, Michaelis-Menten-kinetics were additionally considered for the formation of the minor metabolite 4′-hydroxymidazolam as well to close the overall mass balance^16^ (Figure S2). 1′-hydroxymidazolam is further glucuronidated in phase II metabolism, which was accounted for by a linear clearance process in the liver^17^. Data from previous studies suggest that the glucuronidation processes are rather located in the pericentral zone^1^. However, since no specific data for 1′-hydroxymidazolam glucuronidation was available in mice, only a homogenously distributed hepatic clearance process was considered here. In humans, only a very small fraction of the administered dose is cleared by the kidney, hence renal clearance was neglected for mice as well (Figure S2).

### Torsemide

Concentration profiles for both the parent drug torsemide and the metabolite hydroxytorsemide, were measured and considered for the establishment of the PBPK model for torsemide. Model development was largely based on human information since specific physiological knowledge was not available for mice. In humans, torsemide is metabolized through the enzyme CYP2C9 while 20% are renally secreted^18^. Hydroxytorsemide is further metabolized and then cleared by the kidney^18^. For the mouse PBPK model for torsemide 20% of the dose was also assumed to be renally excreted. The staining data of CYP2C shows enzyme expression in 49% of the liver lobule (Table 1). An additional linear clearance process in the liver was therefore considered for torsemide (Figure S3).

### Codeine

The three main metabolites of codeine in mice are norcodeine, codeine-glucuronide, and morphine, which is further metabolized into morphine-3-glucuronide^19^. Metabolism from codeine to norcodeine is catalyzed by CYP2D22^20^ which is pericentrally expressed in 10% of the liver (Table 1). However, since no PK information was available for the metabolite, only a linear homogenously distributed clearance process in the liver was considered here. Roughly 6.9% of the administered dose was found to be renally secreted unchanged in mice^19^ which was also considered in the model (Figure S4).

### Talinolol

In humans, around 55% of talinolol is excreted unchanged in the urine^21^. In contrast, several metabolites were identified in the urine after talinolol administration in mice^22^. Yet, detailed information of the metabolic pathways is not available in this species. A homogenous distribution of hepatic metabolism was hence assumed and 55% of the administered dose were assumed to be renally excreted in mice as well (Figure S5).

### Pravastatin

In humans, pravastatin is cleared via metabolism as well as via renal and biliary secretion. Around 30% of the administered dose is metabolized, while 41% can be recovered unchanged from the urine and 23% unchanged from the feces^23, 24^. Since equivalent information was not available for mice, the same processes were assumed in the mouse PBPK model as for the human case. The PBPK model hence includes hepatic metabolism, biliary clearance and renal clearance (Figure S6).

### Pharmacokinetics of the drug cocktail in healthy mice

**Figure 4:**
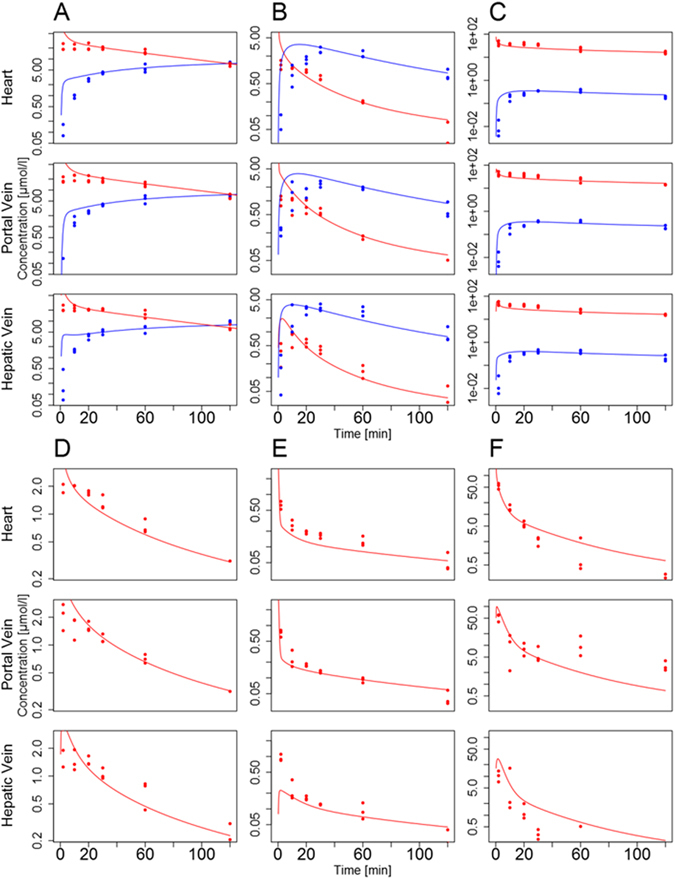
Simulated plasma concentration profiles (lines) for healthy mice and the corresponding experimental data (points) (red: parent drug; blue: metabolite). PBPK simulations are shown for caffeine and paraxanthine (**A**), midazolam and 1′-hydroxymidazolam (**B**), torsemide and hydroxytorsemide (**C**), codeine (**D**), talinolol (**E**), pravastatin (**F**).

Upon establishment of the basic PBPK model structures (Figures S1–S6), the independent model parameters (Tables 2 and 3) were identified by minimizing the deviation between the simulated plasma curves in the portal vein, in the hepatic vein and in the venous blood pool (right heart chamber) on the one hand and the corresponding experimental PK measurements on the other (Fig. 2). Following parameter adjustment, simulations of the PBPK plots show excellent agreement with the experimental data, for both parent drugs and the metabolites considered (mean Pearson correlation: 0.74, mean concordance correlation^25, 26^: 0.51) (Figs 4 and S9 and Tables S2 and S3). In particular, all models simultaneously describe time-concentrations at the three sampling sites such that an adequate level of model quality can be assumed. Highest deviations can generally be observed at early time points. This is probably due to distribution effects between plasma and the surrounding tissues which requires some time for equilibration to be reached *in vivo*. This effect, however, cannot be adequately represented in the PBPK model since the underlying well-stirred assumption implies an immediate equilibrium. For pravastatin, the plasma concentration profile of the portal vein showed much higher concentration values than the ones of the hepatic vein and the heart, which is a clear indication of enterohepatic cycling. Since parameter identification of this process would be impossible from the available PK data, enterohepatic cycling was nevertheless neglected in the PBPK model of pravastatin.

### Pharmacokinetics of the drug cocktail in CCl_4_-treated mice

After establishing the models for the healthy mice, we analyzed the effect of localized CCl_4_-induced damage in the pericentral compartment of the liver lobule on hepatic clearance capacity. For this purpose, a single dose of 1.6 g/kg CCl_4_ was administered i.p. to C57Bl6/N mice in order to destroy the pericentral hepatocytes. 24 h later, when CCl_4_-induced liver damage reaches a maximum, the drug cocktail was injected i.v. and blood samples were collected from the same three sampling sites as described above (*see* Suppl. Data). This allowed on the one hand to quantify the functional loss of drug clearance capacity following CCl_4_ intoxication. On the other hand, a comparative experimental quantification of the dead cell area through immunostaining enabled assessment of the underlying cellular processes (Fig. 3C).

In a first step, we calculated the resulting AUCs (AUC: area under the curve) in the healthy and in the intoxicated case (Table 4). As expected, the AUCs of the parent compounds show a strong increase after CCl_4_-induced liver damage ranging from 44% (torsemide) to 39% (talinolol) which is due to an impaired hepatic clearance of the substance. Vice versa, the AUCs of the metabolites paraxanthine and hydroxytorsemide are reduced by 97% and 71%, respectively. Interestingly, the AUC of 1′-hydroxymidazolam increases by 23% indicating that biotransformation of the metabolite is affected to a larger extent than that of the parent drug in this case.

**Table 4:** Mean AUCs (±SD) [µmol*min/l] of the experimental PK data for the healthy (hlt) and the damaged (tox) case at the three sampling sites.

Drug	Caffeine		Midazolam		Torsemide		Codeine		Talinolol		Pravastatin	
	hlt	tox	hlt	tox	hlt	tox	hlt	tox	hlt	tox	hlt	tox
Hepatic vein	3638 ± 280	5981 ± 227	58 ± 6	256 ± 19	6398 ± 352	9312 ± 722	189 ± 19	340 ± 66	29 ± 3	166 ± 13	249 ± 110	2371 ± 711
Portal vein	3374 ± 194	5431 ± 333	78 ± 7	247 ± 15	6103 ± 433	8496 ± 321	201 ± 11	296 ± 24	29 ± 2	143 ± 11	2154 ± 450	2380 ± 178
Heart	3614 ± 200	5809 ± 265	94 ± 4	299 ± 11	6180 ± 374	9085 ± 582	221 ± 14	298 ± 16	34 ± 3	155 ± 11	1005 ± 114	2217 ± 201

### Functional damage

**Figure 5:**
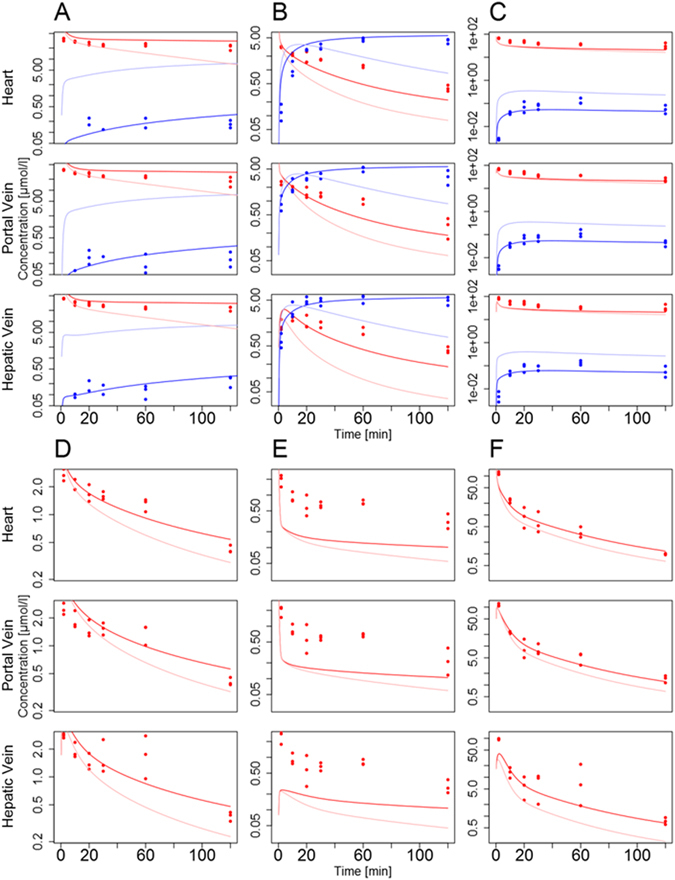
Simulated plasma concentration profiles (lines) for intoxicated mice and the corresponding experimental data (day one after CCl_4_ administration, points) (red: parent drug; blue: metabolite), as well as the simulation of the healthy case (light red and light blue). The models for the damaged case were established by reducing the overall concentration of ADME-associated enzymes and transporters within the liver PBPK models. Simulations are shown for caffeine and paraxanthine (**A**), midazolam and 1′-hydroxymidazolam (**B**), torsemide and hydroxytorsemide (**C**), codeine (**D**), talinolol (**E**), pravastatin (**F**).

The PBPK models for the healthy reference case were subsequently used to estimate the impact of CCl_4_-induced liver damage on hepatic clearance capacity. Notably, all model parameters identified in healthy mice were left unchanged and only the overall concentrations of ADME-associated enzymes were reduced in each PBPK model relative to the healthy reference case such that the resulting PK simulations were again in agreement with the measured data (Table 1 and Fig. 5). Notably, the thus identified reduction in ADME-associated enzyme and transporter concentrations directly reflects the decrease in catalytic activity^12^ and also corresponds to minimal overall model errors as shown in an iterative analysis (Figure S7). Moreover, the relative reduction in hepatic concentrations henceforth referred to as functional damage, is sufficient to explain changes in whole-body plasma PK levels. Following this iterative adjustment of enzyme and transporter concentrations, the simulated PK profiles were again in excellent agreement with measured PK data (Fig. 5; mean Pearson correlation: 0.77, mean concordance correlation^25, 26^: 0.54; Tables S2 and S3 and Figure S10). The identified functional loss varies between 35% (codeine) and 100% (talinolol) relative to the healthy reference case. The fact that adjustment of a single physiological model parameter is sufficient to account for the significant changes in whole-body drug PK can be seen as another strong indicator of structural model validity. This is since the three sampling sites enable an accurate quantification of hepatic clearance capacity, as such ensuring sensitivity of the model behavior with respect to the overall concentrations of ADME-associated enzymes and transporters. Interestingly, the identified functional loss following CCl_4_-induced liver damage is larger than 80% for caffeine, midazolam and torsemide for which the relative pericentral expression had been measured before by immunostaining (Tables 1 and S1). In addition, for talinolol even a full removal of hepatic metabolic activity was not sufficient to explain the observed PK behavior (Figure S7).

### Tissue damage

While the functional damage relates the CCl_4_-induced loss in in hepatic clearance capacity to a reduction in overall enzyme or transporter concentrations and hence in catalytic activity, it should be noted that the functional damage could actually be due to several reasons, i.e. (i) reduction in the mass of liver tissue due to CCl_4_-induced cytotoxicity, (ii) reduction in overall enzyme or transporter concentration in the surviving liver tissue and (iii) further biochemical alterations at tissue level. To further characterize the CCl_4_-induced tissue damage we next determined the loss in viable tissue by H&E-staining. It was found that 36% of liver lobule volume is destroyed. This information, together with protein expression data in the healthy reference mice can be used to estimate the reduction in concentration of ADME-associated enzymes and transporters in the intoxicated case, which is henceforth referred to as *tissue damage* (Table 1, Materials and Methods, Figure S8).

### Comparative analysis of functional damage and tissue damage

A comparison of functional damage and tissue damage next allowed to further analyze the impact of CCl_4_-induced intoxication at tissue level (Fig. 1). If for example both values are equal, it can be assumed that the observed loss in catalytic activity is only due to a reduction in tissue volume. Such an observation can be seen as an uncompensated loss of expressing tissue (*uncompensated loss*). However, during evolution the liver might have established numerous mechanisms to adapt to intoxication. Therefore, it cannot be excluded that the surviving tissue upregulates metabolic pathways to compensate for the lost tissue (*compensated loss*). Vice versa, it is also conceivable that surviving hepatocytes close to a dead cell area lose their metabolic activity due to cell stress (e.g. because of compromised NADPH synthesis). This leads to an aggravated loss of hepatic clearance capacity in response to intoxication (*aggravated loss*). Notably, the comparative analysis of functional damage and tissue damage allows differentiating between the three cases, uncompensated, compensated, and aggravated loss, respectively, in an *in vivo* situation. Interestingly, the estimated damage is significantly smaller than the functional damage identified with the PBPK models in all cases. Hence, the aggravated loss of hepatic clearance capacity clearly indicates that the CCl_4_-induced loss of function goes beyond what could be expected from the pure loss of liver tissue. To support this finding we additionally performed immunostaining of cytochrome P450s in the damaged case (Fig. 3D). This experimental analysis showed that enzyme expression is almost completely vanished after CCl_4_ intoxication. The remaining weak signal is background from dead cells, although the area of most CYPs in healthy livers is larger than the damaged zone. A comparison of functional and tissue damage, respectively, therefore shows that also the tissue that survives the CCl_4_-induced intoxication suffers from functional losses that influence pharmacokinetics.

## Discussion

We here analyzed the impact of CCl_4_-induced damage on hepatic clearance capacity by using an integrative approach of PBPK models and targeted experimental data. To quantify drug clearance *in vivo* a specifically designed drug cocktail consisting of six marketed drugs was used^7^. For each of the six parent drugs and three of the corresponding metabolites PBPK models were developed. Liver zonation was considered in the PBPK models for caffeine, midazolam and torsemide. For these three models, the relative enzymatic distributions of CYP1A, CYP3A and CYP2C in the pericentral and periportal zones of the liver were measured by immunostaining in healthy mice (Fig. 3B and Table 1). This information was integrated into PBPK models with a zonated liver lobule. For model establishment plasma concentration levels of the drugs included in the cocktail were used. Notably, the pharmacokinetic profiles of the six parent drugs and three of the corresponding metabolites were simultaneously measured at three different sampling sites to quantify liver clearance capacity in healthy mice. The consideration of measurements from different sites of the body allows confining the overall mass balance and thus supports parameter identification. After model establishment, the different PBPK models could describe the experimental PK data of all compounds with excellent accuracy (Fig. 4).

The advantages as well as the limitations of the different models are given by the mechanistic nature of the PBPK modelling. On the one hand, physiologically-base modelling allows to model and simulate ADME-related processes within the whole body, however, a sufficient degree of prior knowledge is required on the other hand. In some cases the involved processes are too complex to be correctly modeled or some of the required physiological details are still unknown. This can be seen in two of our models: Enterohepatic cycling in the pravastatin model for example was neglected, since this would have otherwise required the introduction of an additional process whose parameters would have been difficult to identify. For the codeine model, hepatic metabolism was simplified. While there are multiple enzymes and metabolites involved, currently available data and literature knowledge remain insufficient for modelling with adequate accuracy. A single metabolic step was therefore assumed, although this represents a simplification.

Following the initial analyses in healthy control mice, CCl_4_ was injected in healthy mice to investigate the functional impact of pericentral liver damage in whole-body drug PK. In analogy to the healthy reference case, the same PK profiles were measured 24 h after CCl_4_ administration. To quantify the resulting functional loss in hepatic clearance capacity, activity of ADME-associated enzymes and transporters was systematically reduced in the PBPK models such that the agreement between the simulated and the experimentally measured PK profiles is as good as possible (Figure S7). Notably, all model parameters identified in the models of the healthy mice were left unchanged in the intoxicated mice with the exception of the overall concentrations of ADME-associated enzymes and transporters. Again, the PBPK models could simulate the experimental PK measurements 24 h after CCl_4_ administration with excellent accuracy (Fig. 5). The fact that experimental measurements of six parent drugs and three of the metabolites can be simultaneously described at three sampling sites, for healthy and intoxicated mice, is a strong indication of structural correctness of the PBPK models.

This is further supported by the observation that a mere reduction in catalytic activity as such corresponding to a decrease in pericentral enzyme or transporter concentration^12^ is sufficient to describe the significant changes in drug PK as observed for the damaged case. Nevertheless, it should be noted that all PBPK models except for caffeine required some assumptions during model building due to the unavailability of further physiological information. This involves in particular either negligence of extrahepatic metabolization capacity or limiting the renal secretion rate. The assumptions do not necessarily influence the pharmacokinetic behavior but they may impact the overall mass balance. For example for talinolol, only a full removal of hepatic clearance capacity was found to ensure an optimal agreement with the available experimental plasma PK data (Figure S7). This could be an indication that in this case the identified pericentral clearance capacity is too low due to an overestimation of renal clearance in the healthy reference model.

Complementarily to the functional damage, the decrease in hepatic clearance capacity was also estimated experimentally for caffeine, midazolam and torsemide by calculating tissue damage induced by CCl_4_ through H&E-staining. The comparison of the model-derived functional loss and the experimentally measured and estimated tissue damage provided important insights in the consequences of CCl_4_ intoxication *in vivo*. For example, if both values are equal, it can be assumed, that the observed loss in hepatic clearance capacity is sufficiently explained by the loss in enzyme and transporter expressing liver tissue and it is not necessary to consider functional consequences of the surviving tissue. If the functional damage is smaller than the detected tissue damage it is likely that some compensatory adaptation occurred in the surviving liver tissue. Vice versa, if the functional damage even exceeded the tissue damage, this aggravated loss of hepatic clearance capacity indicates that the CCl_4_-induced damage goes well beyond the loss of tissue and also influences the surviving tissue of the liver lobules. Interestingly, we found that the functional loss is generally higher than the tissue damage (Table 1). As discussed above for talinolol, this observation could be due to the fact that the estimated hepatic capacity in the PBPK model is too low. However, it should be noted that for example for caffeine the mass balance could be fully closed for mice, since all physiological information including distribution of metabolizing enzymes and renal excretion were available. Likewise, hepatic clearance capacity for midazolam may rather be seen as an overestimate since CYP3A enzyme expression in other tissues was explicitly neglected. Given the good quality of the different PBPK models, the fact that even a decrease in overall enzyme concentration by more than 90% is needed to describe the PK profiles in the damaged case indicates that CCl_4_-administration affects hepatic clearance capacity to a larger extent as could be explained by the mere loss of pericentral tissue due to cytotoxicity. Hence, based on the dead cell area alone the functional consequences would have been underestimated. This finding was further substantiated by immunostaining of cytochromes which showed almost no remaining enzyme expression in the damaged case (Fig. 3D). Additional physiological alterations in the liver following CCl_4_ administration hence cause the observed decrease in hepatic clearance capacity. For example, it may be possible that CCl_4_-induced liver damage affects blood flow of the liver *in vivo* or NADPH synthesis may be compromised. Such changes could reduce hepatic clearance capacity without cell death of hepatocytes. It is also possible, that CCl_4_-induced liver damage leads to an adaptive enzymatic reprogramming along the sinusoid thereby inducing a further shift in hepatic clearance capacity. The investigation of the underlying biochemical and cellular processes clearly needs further analyses in the future. This should also involve quantification of metabolic capacity in activity assays in in order to correlate enzyme expression in the healthy and intoxicated case beyond immunostaining (Fig. 3B and D).

The combination of targeted experimental measurements and physiological modelling at organism level as applied in this study provides an integrative platform for studies in this field of research. Ideally, experimental analyses could be complemented by advanced test systems^27, 28^. Such integrative studies will help to unravel pathophysiological changes during pathogenesis and disease progression in the future. The here established modeling strategy already provided some important insights into functional consequences of liver damage in the complex situation of a zonated tissue and the differentiation between uncompensated, compensated as well as aggravated tissue destruction that would have been impossible to achieve by conventional methods.

## Methods

### PBPK modelling software

PBPK models for the six cocktail drugs and their corresponding metabolites were developed by using the the free PBPK software PK-Sim® and MoBi v6.0.3 (https://github.com/Open-Systems-Pharmacology). The parameters given in Tables 2 and 3 are sufficient to inform each specific PBPK model in this study and to perform the simulations for the healthy reference case. The concepts underlying PBPK modelling with the software PK-Sim, which was also applied in this study, have recently been described in an instructive tutorial^6^.

### Parameter identification

Independent parameters in the PBPK models were identified by comparing the simulation results to the mean experimental concentration values for each time point and each sampling site. As error function, the least squared function between the observed data and simulated data on a logarithmic scale was used.

1 $$E=\sum _{i}\,\mathrm{log}\,{(\frac{s({t}_{i})}{{\bar{x}}_{i}})}^{2}$$

Here, ${\bar{x}}_{i}$ is the mean observed value at time *t*_*i*_ and s(t_i_) is the simulated concentration. For parameter identification the FME-package (Levenberg-Marquardt-algorithm) of the software R (version 2.5.13) was used.

Since the Levenberg-Marquardt-algorithm is a gradient-based optimization the identification of a global optimum cannot be guaranteed. We hence varied manually the initial estimates of the model parameters in order to obtain good initial guesses for the optimizations. Note, that the model error increases for any alteration in functional damage in Fig. S7 which, together with the correlation coefficients in Tables S2 and S3, indicates that an adequate level of optimality has been reached.

### Animal experiments

8–10 weeks old male C57BL/6N mice, weighing 20–25 g (Charles River, Sulzfeld, Germany) were used. The mice were fed ad libitum with ssniff R/M-H, 10 mm standard diet (ssniff, Soest, Germany) and housed at controlled ambient temperature of 25 °C with 12 h day/12 h night cycle. All experiments were performed in accordance with the relevant guidelines and regulations and approved by the local animal ethics committees (institution name: Landesamt fuer Natur, Umwelt und Verbraucherschutz Nordrhein-Westfalen. Application number: 84–02.04.2012.A333). Acute liver damage was induced by a single intraperitoneal injection of CCl_4_ (1.6 g/kg) diluted in olive oil (1:4). For pharmacokinetic analysis a cocktail of caffeine (5 mg/kg), midazolam (2 mg/kg), torsemide (2 mg/kg), codeine (2 mg/kg), talinolol (1 mg/kg) and pravastatin (20 mg/kg) was injected as intravenous bolus in the tail vein of untreated mice as well as on day one after CCl_4_ administration. Blood samples were collected in a time-resolved manner (2, 15, 30, 60 and 120 min) after administration of the cocktail from the portal vein, the hepatic vein and the right heart chamber as described before^29^. Three mice were used for each time point. Subsequently, blood plasma was separated by centrifugation at 10,000 rpm for 10 min and stored at −80 °C until analysis.

### Quantification of cocktail drugs and metabolites in mouse plasma

Caffeine, paraxanthine, pravastatin, talinolol, torsemide, hydroxytorsemide, midazolam, and 1′-hydroxymidazolam in mouse plasma were quantified by LC-MS-MS as described previously^8^. For determination of codeine, codeine-6-glucuronide, morphine, and morphine-3- glucuronide 40 µl of plasma were spiked with internal standard mixture and diluted with water. Samples were loaded on Isolute C8 100 mg solid phase extraction (SPE) columns (Biotage, Uppsala, Sweden), preconditioned with methanol, water and ammonium carbonate buffer (10 mM, pH 9.3). The SPE columns were washed with ammonium carbonate buffer, dried for 10 min and eluted with methanol. The methanol eluate was evaporated to dryness and the residue dissolved in mobile phase. After centrifugation, the supernatant was used for LC-MS-MS analysis. HPLC separation was achieved on a Synergi Polar column (150 × 2.1 mm I.D., 4 µm particle size, Phenomenex, Aschaffenburg, Germany) using (A) 0.1% formic acid in water, and (B) 0.1% formic acid in acetonitrile as mobile phases at a flow rate of 0.4 ml/min. Gradient runs were programmed as follows: 10% B from 0 min to 1.5 min, linear increase to 50% B to 6 min, increase to 70% B to 6.1 min, remaining at 70% B to 8 min, then re-equilibration.

The mass spectrometer was operated in the multiple reaction monitoring (MRM) mode at a dwell time of 50 ms. MRM transitions and MS parameters were described previously^8^.

Standardization of the analytical assays was performed with calibration samples prepared in plasma in the concentration range from 0.004 to 2 µM for hydroxytorsemide and 1′-hydroxymidazolam, 0.006 to 3 µM for talinolol, 0.008 to 4 µM for midazolam, 0.4 to 200 µM for pravastatin and torsemide, and 0.12 to 60 µM for caffeine and paraxanthine. Calibration samples for codeine and its metabolites were prepared in plasma in the concentration range from 0.0125 µM to 25 µM for codeine, 0.00625 to 12.5 µM for morphine-3-glucuronide, and 0.003125 to 6.25 µM for morphine and codeine-6-glucuronide. Calibration curves based on internal standard calibration were obtained by weighted (1/x) linear regression for the peak-area ratio of the analyte to the respective internal standard against the amount of the analyte. The concentration of the analytes in unknown samples was obtained by linear regression analysis. Assay accuracy and precision were determined by analyzing quality control samples that were prepared like the calibration samples.

### Histopathology

Hematoxylin and eosin (H&E) staining was performed in 5 µm thick formalin-fixed paraffin-embedded liver tissue sections as described before^29^. The dead cell area was quantified using Cell^M^ software (Olympus, Hamburg, Germany) in ten representative images from each mouse.

### Immunohistochemistry

Immunostaining was performed in frozen liver sections (5 µm) using antibodies against CYP3A (Biotrend, Cologne, Germany), CYP1A, CYP2C and Cyp2D (a gift from Dr. R. Wolf, Biochemical Research Centre, University of Dundee, Dundee, United Kingdom). The antibody bindings were identified using appropriate horseradish peroxidase-conjugated secondary antibodies (Dako Denmark A/S, Glostrup, Denmark) and AEC+ high sensitivity substrate chromogen (Dako).

### Image analysis

The immunostaining images contained three visually clearly separable areas: (1) tissue without staining, (2) stained tissue and (3) areas without tissue. The latter represent blood vessels. Due to the variability within the different pictures, manual thresholds were set to each image to identify the three different areas. The percentage *p* of expressed area for enzyme *e* was then calculated by

2 $${p}_{e}=\frac{1}{{M}_{e}}\sum _{i=1}^{{M}_{e}}\frac{{n}_{i}^{s}}{{n}_{i}^{s}+{n}_{i}^{u}}$$

where *M*_*e*_ is the overall number of staining images for enzyme *e*, ${n}_{i}^{s}$ the number of pixels classified as stained tissue and ${n}_{i}^{u}$ the number of pixels classified as not stained tissue on *i*-th picture.

### Calculation of functional damage and tissue damage

To determine the impact of CCl_4_-intoxication on the liver clearance, the overall concentrations of ADME-associated enzymes and transporters in the models of the healthy mice were adjusted to meet the concentration profiles of the intoxicated case. The corresponding value of the functional damage can be described as

3 $$functional\,damage=1-\frac{{e}_{d}}{{e}_{h}}$$

whereby *e*_*h*_ is the amount of enzyme or transporter in the healthy liver and *e*_*d*_ is the adjusted amount of enzyme in the intoxicated liver.

The tissue damage was calculated by using the results of the staining images: The percentage of liver tissue in which an enzyme is expressed (*p*_*e*_) in healthy mice was quantified with immunostaining images. Additionally, the percentage of the liver tissue which gets damaged by CCl_4_ (*d*) was determined by H&E-staining. This value was referred to as tissue damage:

4 $$tissue\,damage=1-\frac{{p}_{e}-d}{{p}_{e}}$$

It should be noted that the calculation of the tissue damage is only based on the loss of active area. Hence two assumptions are implicitly made: (1) enzyme expression is homogenous within the liver tissue and (2) no adaptation occurs in the remaining liver.

## Electronic supplementary material

Supplementary info

Supplementary data
