# Supplementary material for: Physiologically-based modelling in mice suggests an aggravated loss of clearance capacity after toxic liver damage
Source: Sci Rep. 2017 Jul 24;7:6224. doi: 10.1038/s41598-017-04574-z (PMC5524914; doi:10.1038/s41598-017-04574-z)
Supplement: Supplementary file 1 — Supplementary info [file 41598_2017_4574_MOESM1_ESM.doc]

Physiologically-based modelling in mice suggests an aggravated loss of clearance capacity after toxic liver damage

Arne Schenk^1^, Ahmed Ghallab^2,3^, Ute Hofmann^4^, Reham Hassan^2,3^, Michael Schwarz^5^, Andreas Schuppert^1,6^, Lars Ole Schwen^7^, Albert Braeuning^5^, Donato Teutonico^6.8^, Jan G. Hengstler^2^, Lars Kuepfer^6,*^

^1^Joint Research Center for Computational Biomedicine, RWTH Aachen University, Aachen, Germany.

^2^Leibniz Research Centre for Working Environment and Human Factors at the Technical University Dortmund, Dortmund, Germany

^3^Department of Forensic Medicine and Toxicology, Faculty of Veterinary Medicine, South Valley University, Qena, Egypt

^4^Dr. Margarete Fischer-Bosch Institute of Clinical Pharmacology and University of Tuebingen, Stuttgart, Germany.

^5^German Federal Institute for Risk Assessment, Dept. Food Safety, Berlin, Germany

^6^Systems Pharmacology, Bayer AG, Leverkusen, Germany.

^7^Fraunhofer MEVIS, Bremen, Germany

^8^Current address: Clinical PK and Pharmacometrics, Institut de Recherches Internationales Servier, Suresnes, France

^*^corresponding author: lars.kuepfer@bayer.com

# Supplementary Material


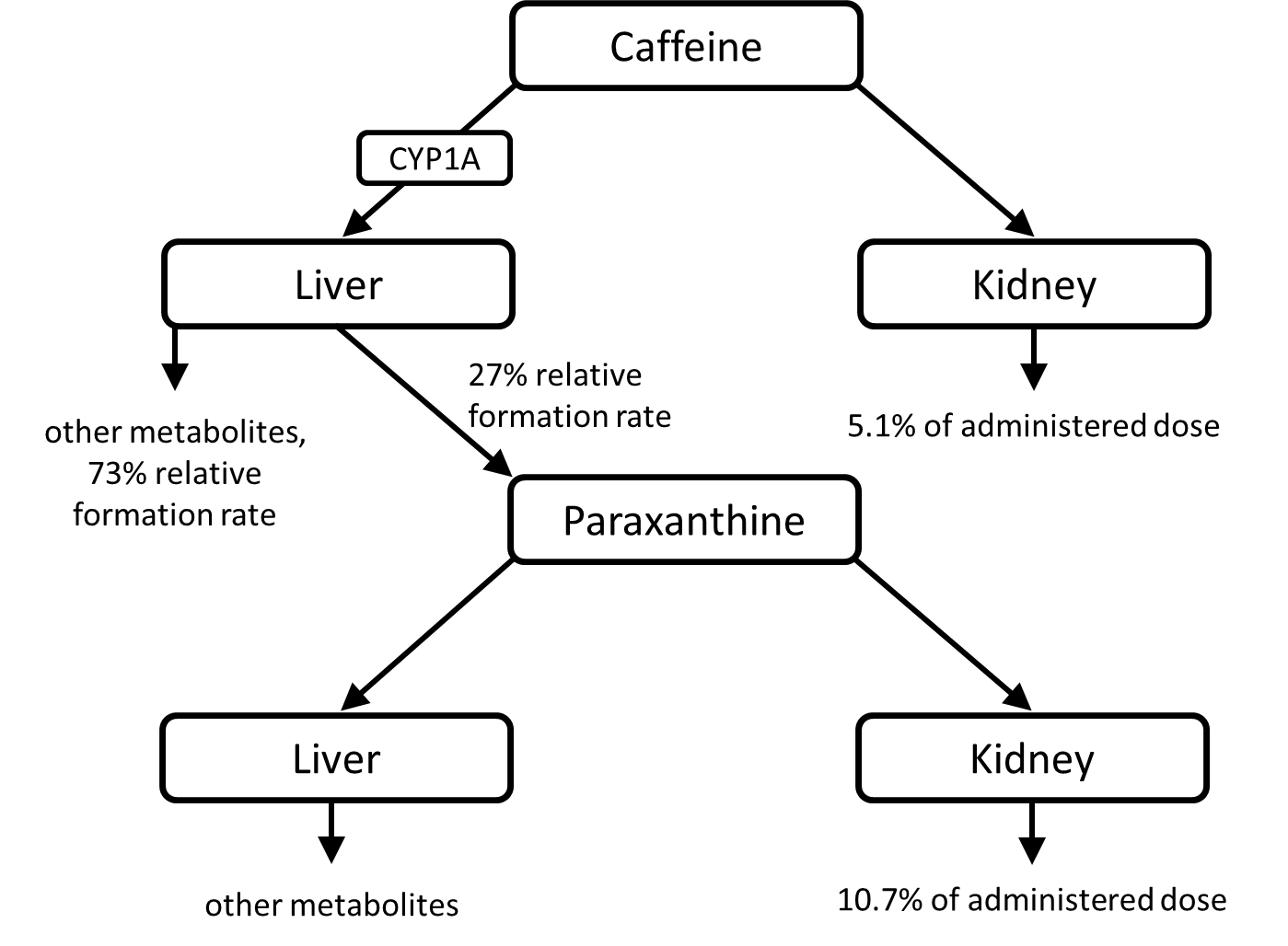


Figure S1: Basic PBPK model structures for caffeine.

#
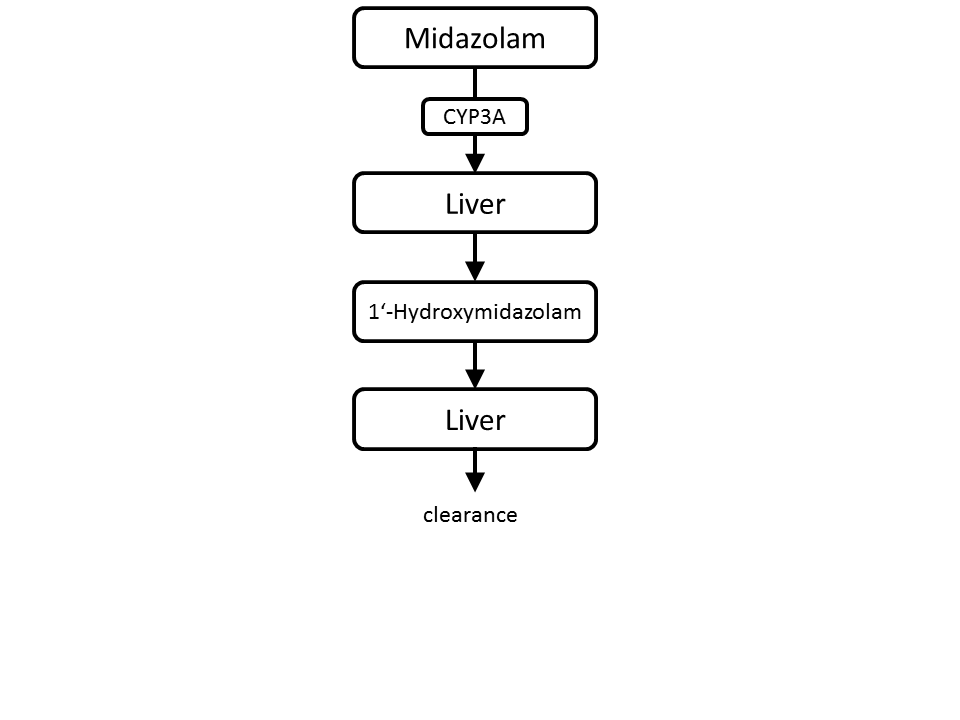


Figure S2: Basic PBPK model structures for midazolam.

#
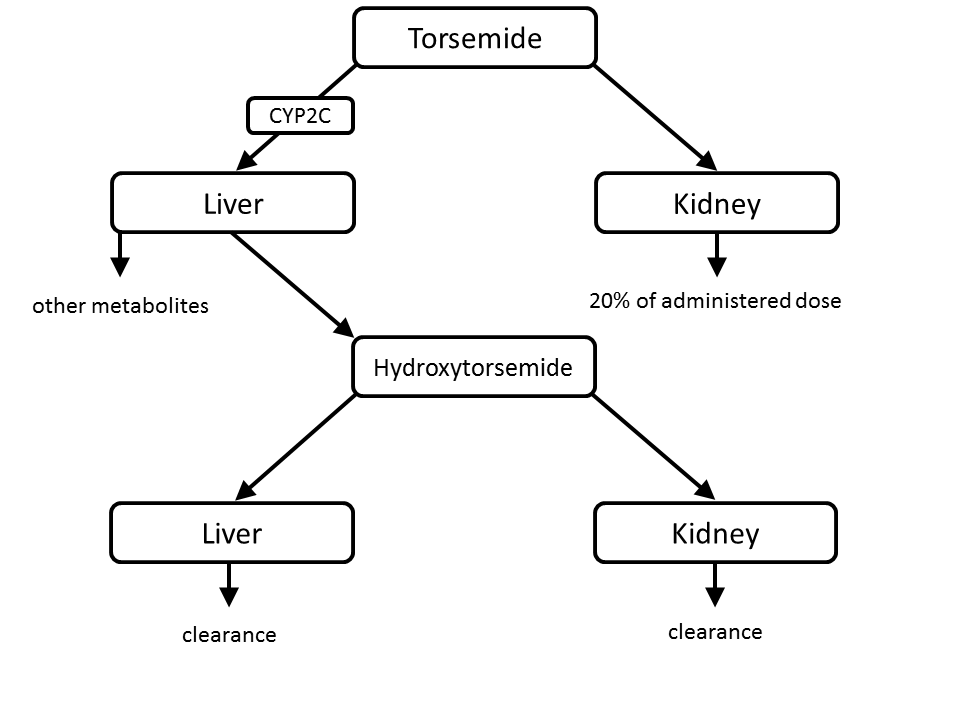


Figure S3: Basic PBPK model structures for torsemide.

#
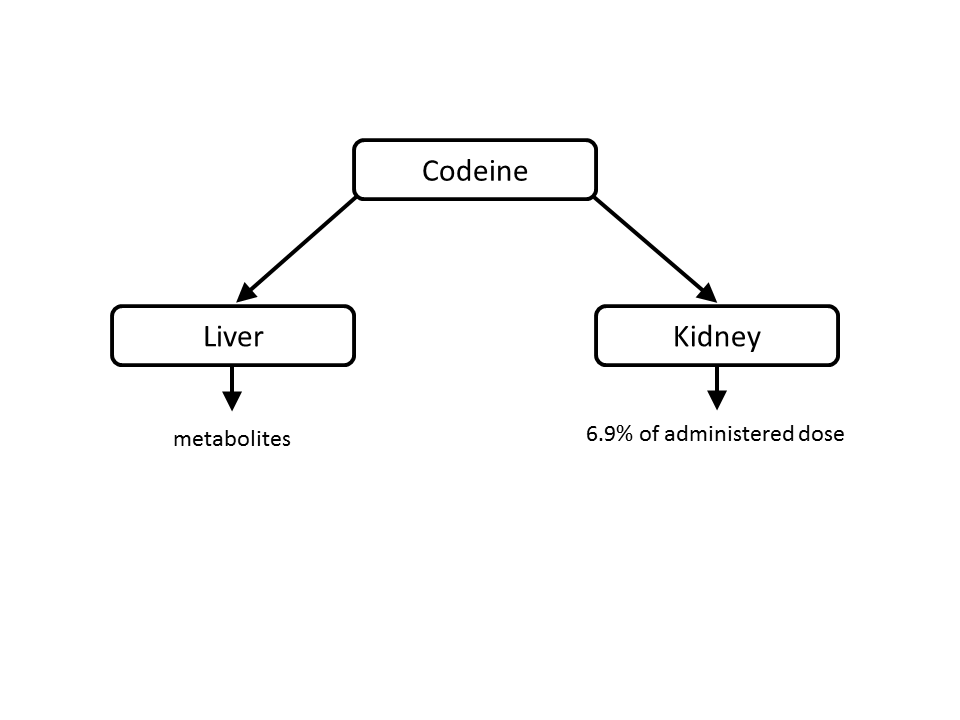


Figure S4: Basic PBPK model structures for codeine.

#
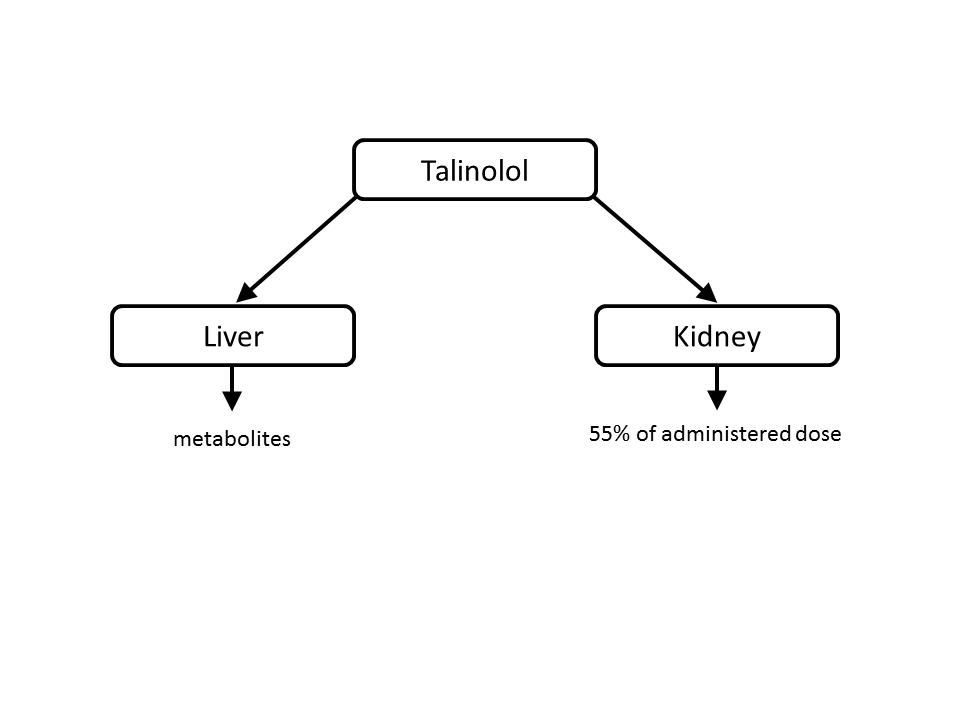


Figure S5: Basic PBPK model structures for talinolol.

#
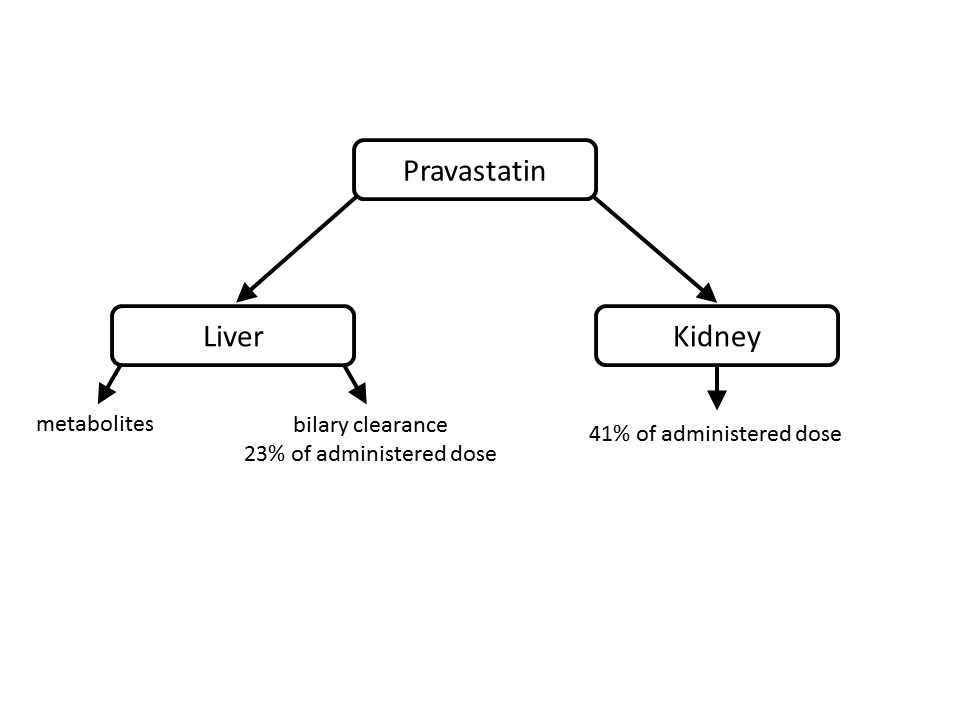


Figure S6: Basic PBPK model structures for pravastatin.

**
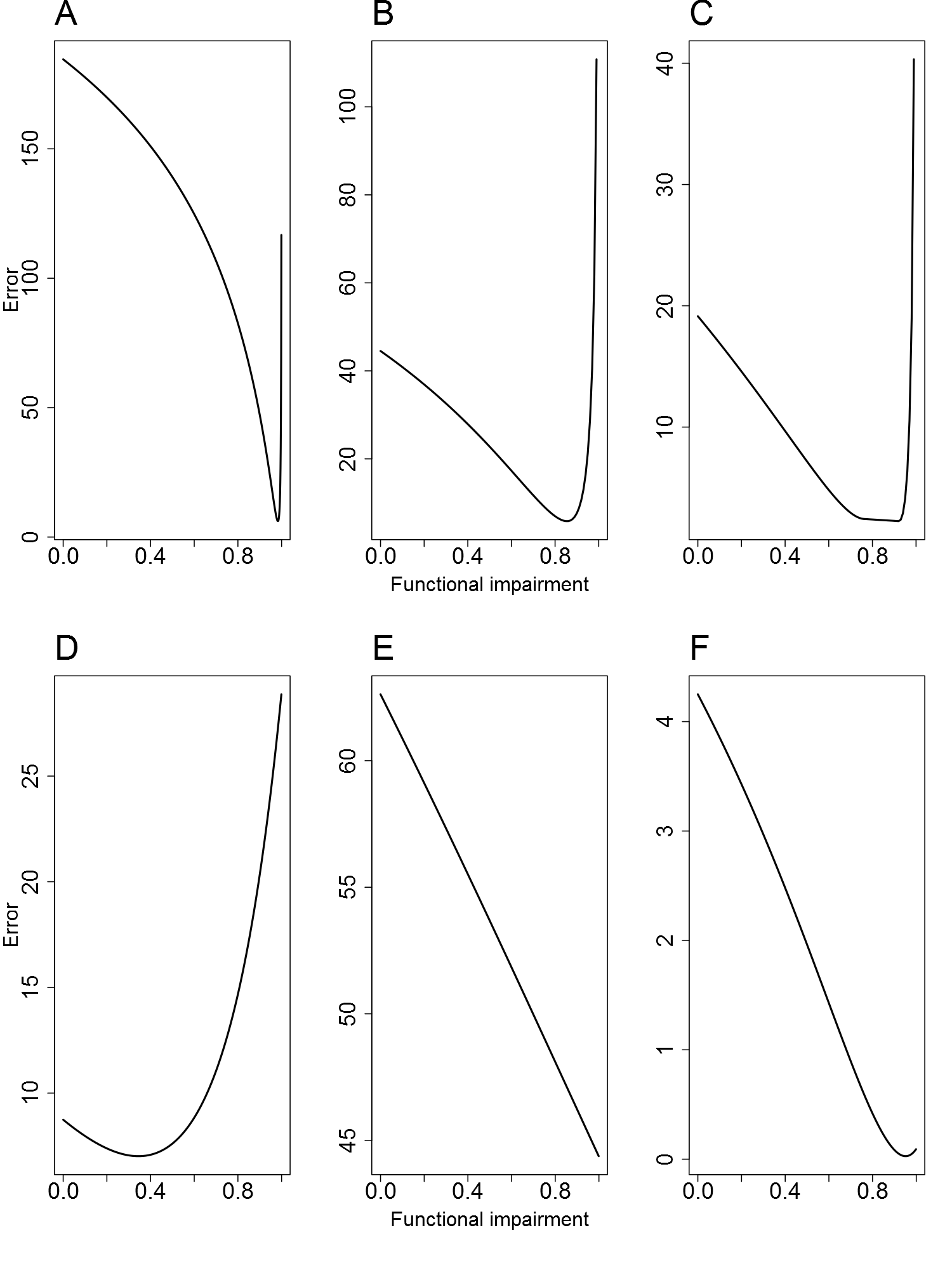
**

Figure S7. Correlating model error and functional damage after CCl_4_-induced intoxication. The error of the model was calculated for different values of functional damage according to Eqn 1. The results illustrate the sensitivity of the functional damage on model accuracy. The results are shown for caffeine/paraxanthine (A), midazolam/1’-hydroxymidazolam (B), torsemide/hydroxytorsemide (C), codeine (D), talinolol (E), pravastatin (F).


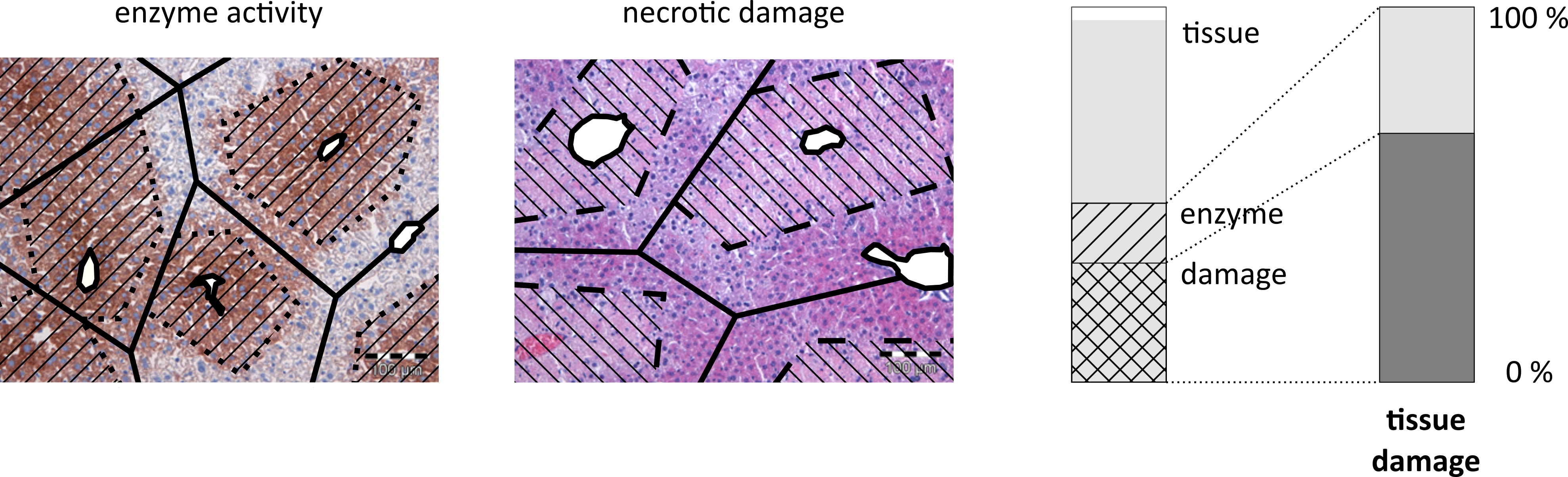


Figure S8.Calculation of tissue damage


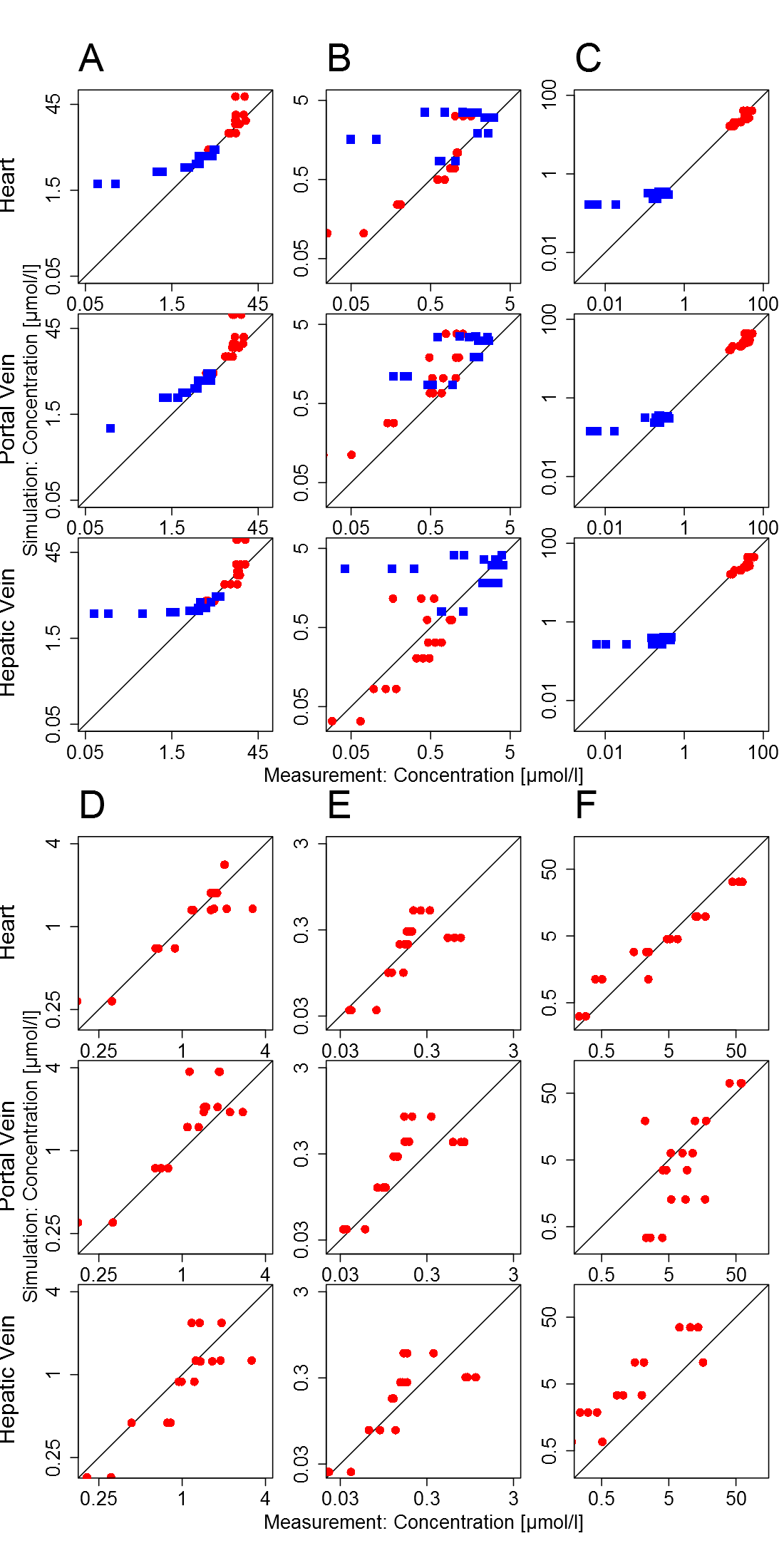


Figure S9. Measured plasma concentration values vs simulated plasma concentration values for healthy mice (red: parent drug; blue: metabolite). The corresponding compounds are caffeine/paraxanthine (A), midazolam/1’-hydroxymidazolam (B), torsemide/hydroxytorsemide (C), codeine (D), talinolol (E), pravastatin (F).


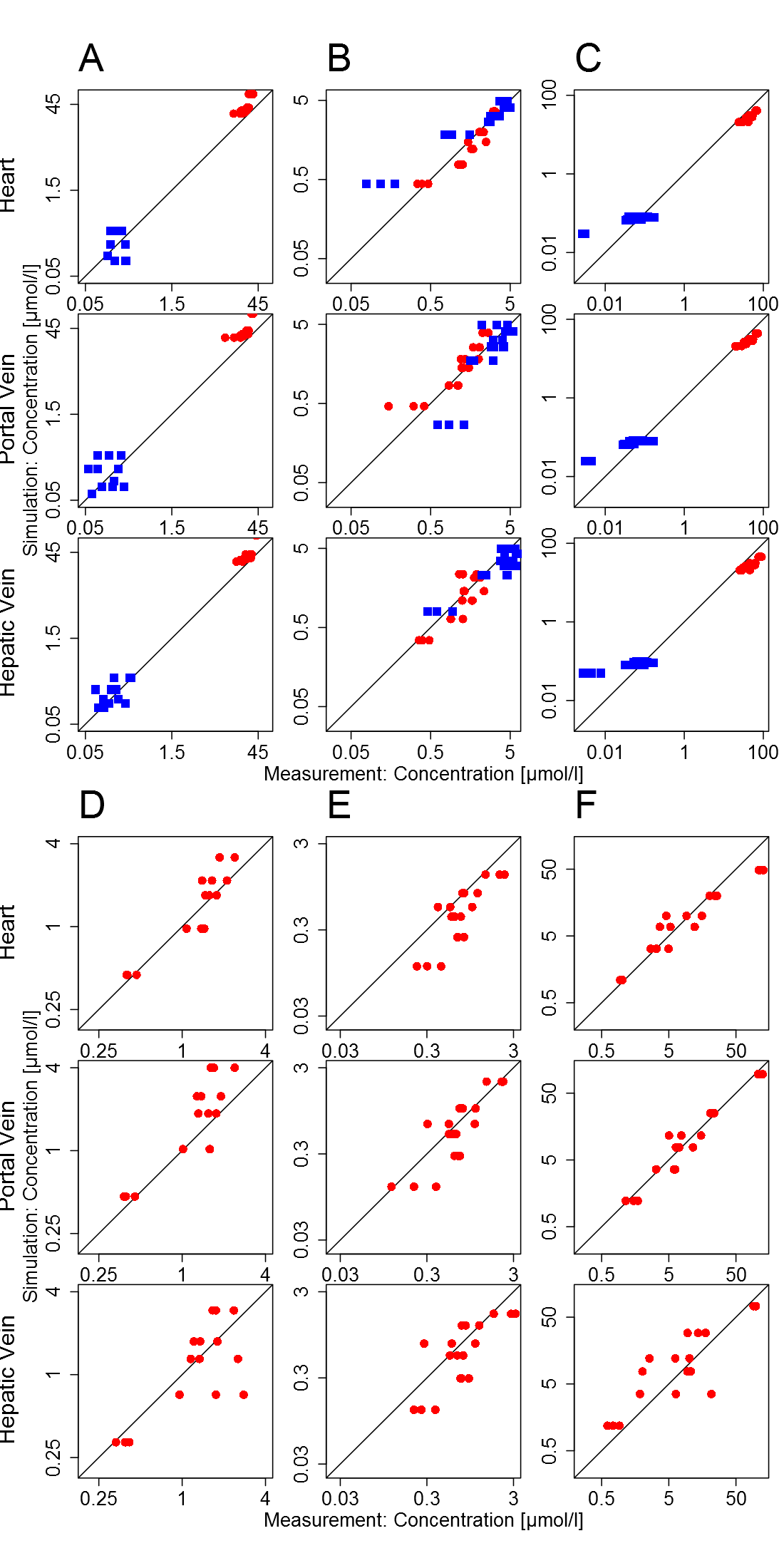


Figure S10. Measured plasma concentration values vs simulated plasma concentration values for intoxicated mice (red: parent drug; blue: metabolite). The corresponding compounds are caffeine/paraxanthine (A), midazolam/1’-hydroxymidazolam (B), torsemide/hydroxytorsemide (C), codeine (D), talinolol (E), pravastatin (F).

Table S1. Functional damage of hepatic clearance capacity of the six cocktail drugs after CCl_4_-induced intoxication.

| **Model** | **Caffeine** | **Midazolam** | | **Torsemide** | | **Codeine** | **Talinolol** | **Pravastatin** |
| --- | --- | --- | --- | --- | --- | --- | --- | --- |
| **Enzyme** | CYP1A | CYP3A | (a) | CYP2C | (b) |  |  |  |
| **Functional damage** | 98.3% | 84% | 99% | 92% | 100% | 35% | 100% | 95% |

Additional clearance for a) 1'-hydroxymidazolam and b) hydroxytorsemide

Table S2. Pearson correlation coefficients between the measured and simulated plasma concentration values.

|  | Healthy | | | Intoxicated | | |
| --- | --- | --- | --- | --- | --- | --- |
|  | Heart | Portal vein | Hepatic vein | Heart | Portal vein | Hepatic vein |
| Caffeine | 0.55 | 0.44 | 0.52 | 0.82 | 0.8 | 0.88 |
| Paraxanthine | 0.97 | 0.94 | 0.93 | -0.09 | 0.03 | 0.6 |
| Midazolam | 0.78 | 0.77 | 0.41 | 0.94 | 0.93 | 0.68 |
| 1’-Hydroxymidazolam | 0.37 | 0.67 | 0.31 | 0.95 | 0.76 | 0.81 |
| Torsemide | 0.76 | 0.76 | 0.82 | 0.93 | 0.93 | 0.91 |
| Hydroxytorsemide | 0.75 | 0.75 | 0.65 | 0.71 | 0.7 | 0.74 |
| Codeine | 0.74 | 0.74 | 0.72 | 0.83 | 0.78 | 0.69 |
| Talinolol | 0.97 | 0.97 | 0.96 | 0.87 | 0.9 | 0.8 |
| Pravastatin | 0.98 | 0.95 | 0.69 | 0.97 | 0.99 | 0.95 |

Table S3. Concordance correlation coefficients between the measured and simulated plasma concentration values.

|  | Healthy | | | Intoxicated | | |
| --- | --- | --- | --- | --- | --- | --- |
|  | Heart | Portal vein | Hepatic vein | Heart | Portal vein | Hepatic vein |
| Caffeine | 0.28 | 0.17 | 0.23 | 0.28 | 0.29 | 0.37 |
| Paraxanthine | 0.85 | 0.88 | 0.64 | -0.07 | 0.02 | 0.57 |
| Midazolam | 0.56 | 0.35 | 0.38 | 0.91 | 0.78 | 0.66 |
| 1-Hydroxymidazolam | 0.23 | 0.44 | 0.27 | 0.94 | 0.74 | 0.69 |
| Torsemide | 0.63 | 0.66 | 0.66 | 0.32 | 0.43 | 0.34 |
| Hydroxytorsemide | 0.53 | 0.58 | 0.35 | 0.5 | 0.53 | 0.44 |
| Talinolol | 0.69 | 0.47 | 0.86 | 0.7 | 0.89 | 0.7 |
| Codeine | 0.37 | 0.21 | 0.37 | 0.37 | 0.22 | 0.38 |
| Pravastatin | 0.9 | 0.78 | 0.31 | 0.62 | 0.97 | 0.91 |
